# Improving the Knowledge and Competency of UK Foundation Doctors in Nasogastric Tube Placement: A National Study

**DOI:** 10.7759/cureus.54434

**Published:** 2024-02-19

**Authors:** Wenyi Cai, Spencer Probert, Sai Y Pendyala, Constantinos Lipsos, Freya Wadey, Muhammad Rafaih Iqbal

**Affiliations:** 1 General Surgery, Mid and South Essex NHS Foundation Trust, Basildon, GBR

**Keywords:** ng tube, medical education, training, foundation doctor, nasogastric tube

## Abstract

Objective

This study aims to improve foundation doctors' knowledge of guidelines for confirming nasogastric (NG) tube position and to enhance their confidence and competency in NG tube placement.

Methods

A three-part educational approach was designed, which included an educational leaflet and allowed the assessment of a participant's knowledge of guidelines pertaining to NG tube positioning before and after education.

This educational leaflet and accompanying pre- and post-learning assessments were distributed among NHS Foundation Trusts in the UK between January 2022 and June 2022.

All participants were foundation doctors in the UK. Those who had entered further training after the completion of their foundation training, at the time of assessment distribution, were excluded.

Results

A total of 173 participants completed this assessment. We found a significant increase in confidence among participants following the education (p<0.05). There was also a significant improvement in objective knowledge of guidelines on NG tube position confirmation following education (p<0.05).

Conclusions

Current knowledge on NG tube positioning is lacking among foundation doctors, but this can be significantly improved with simple educational leaflets. Furthermore, many participants felt that more training is needed, and this topic should be included in an essential teaching program.

## Introduction

Since the invention of the nasogastric (NG) tube in the early 20th century, it has become an indispensable tool in modern medical practice. NG tube insertion is one of the most common medical procedures performed at a patient's bedside [1]. The procedure involves introducing a flexible tube through the nasal cavity, and then advancing it down the esophagus into the stomach. NG tubes have two primary purposes: the administration of medications and feeds, and the decompression of the upper GI tract in cases of obstruction [2].

Despite NG tube insertion being a common medical procedure, malposition occurs frequently. The administration of feed, fluid, and medication into an intrapulmonary NG tube and the complications that result thereafter are considered a “Never Event” in the United Kingdom (UK) [3]. A “Never Event” is defined as a medical incident that is “fully preventable” under the guidance and safety recommendations available at a national level [3]. Complications from the use of a malpositioned NG tube include aspiration, pneumothorax, and esophageal perforation, all of which can lead to fatal outcomes [1].

The national guidance on confirming NG tube position is set out by the National Patient Safety Agency (NPSA) in the UK [4]. Currently, testing the pH of gastric aspirate is considered the first-line investigation for confirming the NG tube position [5]. The NG tube is considered safe to use if the pH value is less than 5.5 [5]. If gastric aspirate cannot be obtained, or the pH is above 5.5, or if gastric pH is made unreliable due to pH-altering medications, such as proton pump inhibitors, antacids, and histamine H2-receptor antagonists, a chest X-ray (CXR) is used to visually confirm the NG tube position [5]. Other methods of confirming NG tube position, such as the whoosh test (auscultating for a gurgling sound over the epigastrium while injecting air into the NG tube), litmus paper, and visual assessment of the color of NG aspirate [6], have extensive disadvantages that have been discussed in NG tube-related literature and have therefore been deemed unsafe by the NPSA [4,5,7].

The use of a malpositioned NG tube first became a “Never Event” in the UK in 2005, as a result of several NG tube-related deaths that occurred in the early 2000s [8]. However, this “Never Event” continues to occur nationwide, and its incidence has not significantly decreased over time [3]. NHS England Never Events data revealed that from April 1, 2015, to September 30, 2021, there were a total of 196 NG tube-related “Never Events,” with an average of 28 incidents per calendar year [9]. All the data generated/analyzed in this study is included in the published article and supplementary files.

Since NG tube insertion is such a common procedure, doctors of all grades are expected to develop competency and confidence in methods of insertion and confirmation. In UK hospitals, newly qualified doctors, known as foundation doctors, are usually the first port of call for nursing staff to confirm NG tube position. As these doctors are in a national training program, they are expected to be taught while on their clinical placements. Identifying learning needs at this level can then aid in shaping learning curricula for subsequent cohorts. This study aims to assess and improve foundation doctors’ knowledge and competency in ascertaining NG tube placement.

## Materials and methods

A three-part educational approach was devised, incorporating an educational leaflet and Google Forms for assessment. Part one consisted of a 16-item assessment, which included a combination of binary choice questions (Yes/No) and multiple-choice questions. These questions collected information on individual participants' demographics, such as their training grade and place of work, as well as their knowledge of NG tube placement confirmation as set out by the NPSA guidelines. The second part provided education on NG tube placement confirmation in the form of a leaflet. Participants could only proceed to part two after fully completing part one, and it was not possible to return to previously submitted answers. This ensured that no participant had access to the educational leaflet before completing part one. Part three consisted of a 6-item assessment, which reassessed participants' knowledge of NG tube placement confirmation following the education, using multiple-choice questions. Both part one and part three also included a question asking individuals to rate their confidence in confirming NG tube placement, assessed with a 10-point Likert scale [10]. The educational leaflet is included in the supplementary data.

This educational leaflet and accompanying pre- and post-learning assessment were distributed among foundation doctors across the UK. Methods of distribution included contacting foundation doctor administrators and foundation doctor representatives and disseminating the leaflet via emails and social media. The assessment was open for six months. All responses have been collected and analyzed in Microsoft Excel. A Mann-Whitney U test was used to assess the significance between the pre-education and post-education confidence levels in confirming NG tube placement. Fisher's exact test was used to determine any significance between the answers to the pre-education and post-education multiple-choice questions.

Ethics approval from the local research committee was not required for this study, as there was no identifiable information in the data collected. This was also confirmed by the NHS Research Ethics Committee tool provided by the Medical Research Council [11]. Informed consent was obtained from all participants for their data to be used anonymously in this study. This was clearly stated in the introduction paragraph of the assessment before any data input was required, giving participants the option to continue or stop. All study methods were performed according to the relevant guidelines and regulations.

## Results

A total of 173 foundation doctors completed the assessment between January and June 2022 (January 1, 2022 to June 30, 2022). Of these participants, 103 (60%) were foundation year one doctors, 56 (32%) were foundation year two doctors, and the remaining 14 (8%) had finished foundation training but had not yet entered further specialist training. Almost all participants who completed the assessment (97%) worked in England, while the remaining 3% were equally divided between Scotland and Wales. Table 1 shows a breakdown of the different regions in which participants worked.

**Table 1:** Distribution of study participants according to their regions of work in the UK.

Regions of the UK	n (%)
East of England	74 (42.8)
London	30 (17.3)
North East of England	19 (11.0)
South West of England	14 (8.1)
Yorkshire and Humber	13 (7.5)
North West of England	8 (4.6)
Midlands	7 (4.0)
Scotland	3 (1.7)
Wales	3 (1.7)
South East of England	2 (1.2)

Most participants (71%) had been asked to validate the correct positioning of an NG tube on a CXR, but only 36% felt confident that they could correctly verify the NG tube position without senior support.

**Figure 1:**
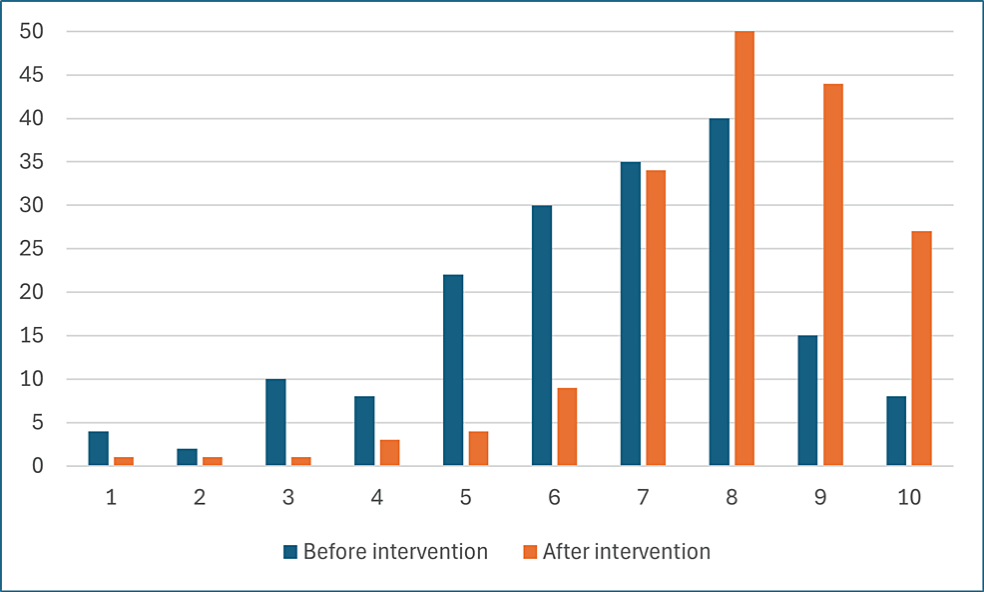
Distribution of confidence rating on a 10-point Likert scale among participants before and after education.

Participants were asked to rate their confidence in identifying the correct position of an NG tube pre- and post-education on a 10-point Likert scale (1 being the least confident and 10 being the most confident). Before the education leaflet, confidence ranged between 1 and 10 with a median of 7 and an interquartile range of 3. Following the education, confidence ratings again ranged between 1 and 10; however, we found a median of 8 and an interquartile range of 2. The distribution of the confidence ratings before and after education can be seen in Figure 1. These results show a significant improvement in confidence following the educational leaflet (p<0.05, Mann-Whitney U test).

Participants were asked to select the correct first- and second-line methods from a list of provided options for confirming NG tube position as set out by the NPSA. For the first-line method, 128 participants (74%) selected the correct option before education, while 167 participants (97%) chose correctly after education. This shows significant improvement (p<0.05, Fisher’s exact test). For the second-line method, 143 participants (83%) answered correctly before education, which increased to 171 participants (99%) after education, again demonstrating a significant improvement in knowledge (p<0.05, Fisher’s exact test).

In the post-education assessment, 130 participants (75%) expressed their wish to receive more training to increase their NG tube competency, and 168 participants (97%) indicated that training on NG tube positioning should be an essential part of the UK foundation programme training.

## Discussion

Our study has shown that knowledge on methods to confirm NG tube position is generally lacking among foundation doctors. However, this can be substantially improved with simple measures such as educational leaflets. Moreover, the lack of confidence in commencing feeding, especially out of hours, may adversely affect patient outcomes, as this may delay the administration of essential medications and nutrition.

This study's intervention, in the form of a simple educational leaflet, has led to significant improvement in both the subjective measure of confidence and the objective measure of NG tube placement knowledge. As alluded to earlier, serious incidents related to NG tube misplacement continued to occur after the NPSA first issued its alert in 2005. Realistically, NG tube-related incidents may be more prevalent than the data suggests, as many incidents are likely unreported. Despite a higher level of awareness among medical professionals, it seems that intervention in the form of a national alert regarding NG tube-related complications has done relatively little to reduce its prevalence.

Yardley IE and Donaldson LJ found that many of the reported incidents, after the issue of the national alert, involved using outdated methods of confirming the NG position or, in some instances, no checks at all [8]. Another reason for the ongoing high prevalence may be attributed to the unreliability of current methods [8,12]. Even for the gold-standard first- and second-line tests (pH testing and CXR, respectively), results can be difficult to interpret with the use of certain medications (PPIs) and variations in esophageal anatomy [8].

Studies have found that awareness of how errors are made and the complications resulting from these errors have a great impact on reducing the future incidence of the errors in question. A lack of awareness of possible errors and their complications may result in a 'blind spot,' giving rise to the continuation and possible propagation of these errors [13]. As we have shown, a simple educational leaflet was enough to significantly improve knowledge in confirming the NG tube position. A national rollout of targeted education would create room for a future study looking for a reduction in the national incidence of NG-specific 'Never Events.' While we believe that our educational leaflet would benefit all grades of clinicians, it has been shown that confidence and competence in clinical skills need to be built in the early stages of a medical career, due to the increasing responsibilities and commitments as one progresses through their career [14]. It is also incredibly valuable to aim this education at the most junior clinicians because, as stated previously, these individuals are often tasked with confirming NG tube positions.

Regarding our study, we have identified a few limiting factors. Firstly, we note a small population size relative to the total number of foundation doctors in the UK. Secondly, we note a bias toward London and the East of England in regard to participant numbers. We attribute this to the author's location, as the majority of our authors worked in these two regions, meaning uptake was more easily achieved in these regions. We believe both of these factors may be improved in subsequent studies by recruiting more contributors throughout the UK with access to different regions. Finally, while participants reported improved confidence in confirming NG placement, we were unable to assess an improvement in actual skills, and this may form the basis of future studies.

## Conclusions

Possessing the competency and confidence to confirm an NG tube position is an essential clinical skill for doctors at all levels of seniority. Competence in confirming the correct placement of an NG tube is vital, ensuring that patients can receive their nutrition, medications, or gastrointestinal decompression in a timely manner, with a reduction in the frequency of adverse events. We have shown that simple educational interventions can be sufficient to significantly improve both confidence and competency in confirming placement. We recommend reinforcing learning at the undergraduate level and providing further education for junior clinicians. Furthermore, by having local guidelines, institutions may be able to prevent the occurrence of these "never events" by providing specific templates for the documentation of NG placement. This would ensure that all necessary documentation is present and can be checked, as well as providing the necessary steps to ensure that NG placement is appropriately confirmed.
